# Pathologic and Immunohistochemical Evidence of Possible Francisellaceae among Aborted Ovine Fetuses, Uruguay

**DOI:** 10.3201/eid2901.220698

**Published:** 2023-01

**Authors:** Federico Giannitti, Matías A. Dorsch, Carlos O. Schild, Rubén D. Caffarena, Karen Sverlow, Aníbal G. Armién, Franklin Riet-Correa

**Affiliations:** Instituto Nacional de Investigación Agropecuaria, La Estanzuela, Colonia, Uruguay (F. Giannitti, M.A. Dorsch, C.O. Schild, R.D. Caffarena, F. Riet-Correa);; University of California, Davis, California, USA (K. Sverlow, A.G. Armién);; Universidade Federal da Bahia, Ondina, Salvador, Brazil (F. Riet-Correa)

**Keywords:** bacteria, zoonoses, Francisellaceae, tularemia, abortion, emerging diseases, pathology, sheep, South America, Uruguay

## Abstract

The only genus of the Francisellaceae family known to contain species pathogenic to mammals is *Francisella*, for which reported cases in the Southern Hemisphere have been limited to Australia. We describe severe necrotizing and inflammatory lesions and intralesional immunohistochemical identification of *Francisella* sp. lipopolysaccharide among aborted ovine fetuses in Uruguay.

The Francisellaceae family comprises gram-negative coccobacilli and 4 genera are currently recognized: *Francisella*, *Allofrancisella*, *Pseudofrancisella*, and *Cysteiniphilum* (*1*), of which only *Francisella* is of clinical relevance. *Francisella tularensis* is the most studied species because it causes tularemia, a highly transmissible, potentially life-threatening, zoonotic disease, also considered a potential bioterrorism agent (*2*,*3*).

Tularemia occurs over almost the entire Northern Hemisphere but is rarely reported in the Southern Hemisphere, where the only published cases have occurred in Australia (*4*–*7*). *F. tularensis* comprises 4 subspecies, *tularensis*, *holarctica*, *mediasiatica*, and *novicida*. *F. tularensis* subsp. *tularensis* occurs almost exclusively in North America and is responsible for 80%–90% of the tularemia cases, despite the co-existence of subspecies *holarctica*, which is the cause of most tularemia cases in Europe (*2*). The few cases of *F. tularensis* infection described in Australia were associated with *F. tularensis* subsp. *holarctica* and *novicida* (*4*–*6*). In the Americas, tularemia occurs in the United States, Mexico, and Canada (*2*,*8*), and no disease caused by *Francisella* spp. bacteria in mammals has been reported south of Mexico.

Although *F. tularensis* has a broad host range, sheep are the only livestock species affected by epizootics of tularemia and have been implicated in disease transmission to humans (*9*,*10*). We report a case of ovine abortion in Uruguay that raises concerns about the possible occurrence of tularemia in South America.

## The Study

**Figure 1:**
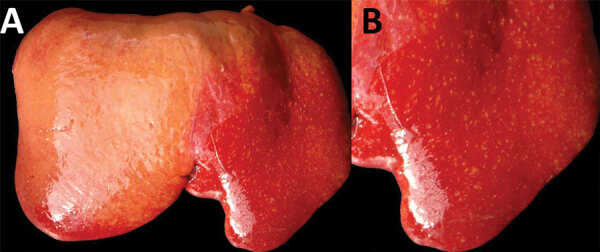
Diaphragmatic view of diseased liver from an aborted ovine fetus (fetus B) with possible Francisellaceae infection, Uruguay. A) Myriad discrete, white to yellowish, round, coalescing foci are visible, ranging from pinpoint to ≈2 mm in diameter, with a multifocal disseminated distribution in the hepatic parenchyma indicative of necrotizing hepatitis. Lesions are more visible in the left liver lobe (right side of the image) than the right liver lobe (left side of the image), in which the hepatic parenchyma is diffusely pale due to moderate autolysis. B) Isolated view of left liver lobe with disseminated foci of necrotizing hepatitis, which are characteristic in cases of tularemia.

**Figure 2:**
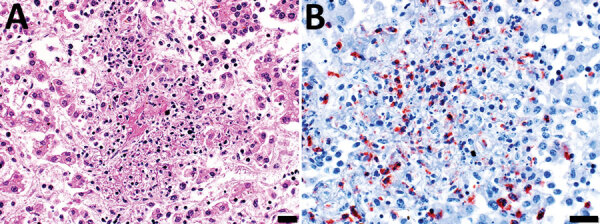
Microscopic images of diseased liver from an aborted ovine (fetus B) with possible Francisellaceae infection, Uruguay. A) Hematoxylin and eosin stain of section of liver. Hepatic histoarchitecture in center of image is effaced by eosinophilic karyorrhectic cellular debris (i.e., necrosis), fibrin exudate, and inflammatory cell infiltrates, mostly neutrophils and macrophages, evidence of severe necrotizing fibrinosuppurative hepatitis. Scale bar indicates 20 μm. B) Immunohistochemistry using a mouse monoclonal antibody raised against *F. tularensis* lipopolysaccharide and hematoxylin counterstain of section of liver shows abundant intralesional intracellular and extracellular immunoreactivity as a granular brownish-red precipitate (chromogen 3-amino-9-ethylcarbazole). Intracytoplasmic immunoreactivity was noted in infiltrating neutrophils and macrophages. Scale bar indicates 20 μm.

In July 2015, two (≈1%) of ≈200 pastured sheep on a family farm in Colonia, Uruguay, aborted at ≈4 months of gestation. Autopsies on twin aborted fetuses (A and B) showed similar gross lesions (Table 1), consisting of severe multifocal widespread necrotizing hepatitis (Figure 1), and moderate fibrinous peritonitis and pericarditis. Samples of liver, adrenal gland, spleen, lung, heart, kidney, and brain tissues of both fetuses were fixed in formalin, then processed, embedded in paraffin, microtome-sectioned, and stained with hematoxylin and eosin. Histopathologic examination revealed severe acute multifocal random fibrinonecrotizing neutrophilic and histiocytic hepatitis (Figure 2, panel A), multifocal necrotizing and neutrophilic myocarditis, multifocal neutrophilic bronchiolitis and alveolitis, and multifocal fibrinous splenic capsulitis.

**Table 1:** Autopsy findings in twin ovine fetuses with possible Francisellaceae infection, Uruguay

Autopsy findings	Fetus A	Fetus B
Crown-to-rump length	30 cm*	32 cm*
Sex	F	F
External aspects	Fully formed with complete wool, hair coat	Fully formed with complete wool, hair coat
Pulmonary aeration	Unexpanded, unventilated lungs†	Unexpanded, unventilated lungs†
Eponychium	Intact (uneroded) in all limbs†	Intact (uneroded) in all limbs†
Abomasum	No colostrum or milk curds†	No colostrum or milk curds†
Umbilical cord	Intact, wet, no hemorrhages or clots in umbilical vessels†	Intact, wet, no hemorrhages or clots in umbilical vessels†
Subcutaneous edema	Yes	No
Fibrinous peritonitis and pericarditis	Yes	Yes
Liver lesions	Enlarged liver with rounded edges, myriad discrete white to yellowish foci <2 mm in diameter disseminated throughout the hepatic parenchyma and visible from the capsular surface	Enlarged liver with rounded edges, myriad discrete white to yellowish foci <2 mm in diameter disseminated throughout the hepatic parenchyma and visible from the capsular surface
Placenta	Not available for examination	Not available for examination
Tissue autolysis	Moderate	Moderate

*Consistent with a gestational age of ≈4 mo. †These findings suggest that the fetuses were dead at the time of expulsion (abortion).

We processed formalin-fixed paraffin-embedded sections of liver from fetus B for immunohistochemistry to detect *Francisella* antigen (Appendix). We used tissue from a squirrel with culture- and PCR-confirmed *F. tularensis* septicemia as a positive control. We performed antigen retrieval in a decloaking chamber by using Antigen Decloaker citrate buffer (Biocare Medical, https://biocare.net). We applied a specific mouse monoclonal IgG3 raised against *F. tularensis* lipopolysaccharide, *F. tularensis* LPS Monoclonal Antibody (T14) (Thermo Fisher Scientific, https://www.thermofisher.com) as the primary antibody at 1:1,000 dilution. We used Mouse-on-Farma HRP-Polymer (Biocare Medical) and 3-amino-9-ethylcarbazole (Thermo Fisher Scientific) for antigen detection. For negative controls, we replaced *F. tularensis* monoclonal antibody with normal mouse IgG for both ovine and squirrel reactions.

Immunohistochemistry revealed strong abundant intralesional granular immunoreactivity in the necrotic foci of the fetal ovine liver, which was largely intracytoplasmic in infiltrating neutrophils and macrophages (Figure 2, panel B). We observed immunoreactivity in the positive control squirrel tissue but not in the negative controls. We conducted ancillary testing to rule out other ovine abortifacients (Table 2).

**Table 2:** Ancillary testing performed in formalin-fixed paraffin-embedded sections of liver of aborted ovine fetus with possible Francisellaceae infection, Uruguay*

Tests	Results
Stains	
Steiner silver	No intralesional curved bacilli (i.e., *Campylobacter*) or spirochetes (i.e., *Leptospira*, *Flexispira, Helicobacter*) detected
Gomori’s methenamine silver	No intralesional fungi detected
Gram	No intralesional bacteria detected
Immunohistochemistry	
*Chlamydia* spp.	Negative
* Coxiella burnetii*	Negative
*Salmonella* spp.	Negative
* Listeria monocytogenes*	Negative
* Toxoplasma gondii*	Negative

*All test results were validated using adequate positive/negative control tissues in each run.

We postfixed formalin-fixed sections of liver from fetus B in modified Karnovsky’s fixative, 1% osmium tetroxide, and 0.1 mol cacodylate buffer, then processed and embedded sections in resin for transmission electron microscopy. Despite suboptimal ultrastructural tissue preservation due to autolysis, intrahistiocytic and extracellular ≈0.7–1.7 μm gram-negative coccobacilli colocalized with the foci of necrotizing hepatitis.

The lack of historical reports of tularemia outside endemic areas of North America and Eurasia has been puzzling (*6*). Recently, tularemia emerged in Australia (*3*) and reemerged in the Northern Hemisphere (*7*). South America has been considered free of tularemia (*7*); a status that seems to be based solely on the lack of disease reporting. However, tularemia might have been undiagnosed because of limitations in disease surveillance systems in the region. No clinical disease caused by *Francisella* spp. in mammals in the Americas south of Mexico has been described. Our results raise concerns about the possible occurrence of tularemia in South America.

The abortifacient effects of *F. tularensis* in sheep have been described in the United States, and tularemia has been regarded as an overlooked syndrome in sheep (*9*). From a pathologic viewpoint, necrotic foci in the liver, spleen, or lungs in late term aborted ovine fetuses are characteristic of tularemia and should raise suspicion, although gross lesions can be absent even in cases with typical histologic inflammatory and necrotizing lesions (*9*). Contrary to most bacterial abortifacients of sheep (*11*), *F. tularensis* is not visible upon histopathologic examination of tissues stained with hematoxylin and eosin, Steiner silver, or Gram stains, even in tissues that have a high bacterial burden demonstrated by immunohistochemistry (*9*). The ultrastructural demonstration of intracellular gram-negative coccobacilli of the expected size in phagocytic and inflammatory cells in tissues with lesions, as in our case, aids in the diagnosis. Diagnostic investigation of any case of ovine abortion with fetal lesions indicating a bacterial etiology should include ancillary testing to identify *F. tularensis* and rule out other abortigenic pathogens (*11*).

The etiologic diagnosis in our case was reached by the immunohistochemical demonstration of abundant intralesional antigen by a specific monoclonal antibody raised against *F. tularensis* lipopolysaccharide. Immunohistochemistry has proven useful for identifying *F. tularensis* in diagnostic settings (*9*,*12*). *F. tularensis* lipopolysaccharide is a main specific antigen and virulence factor and differs from the lipopolysaccharide of other gram-negative bacteria (*13*). According to the manufacturer, the primary antibody we used for immunohistochemistry does not cross-react with *F. tularensis* subsp. *novicida*, *Yersinia pestis*, *Y. pseudotuberculosis*, *Y. enterocolitica*, *Vibrio cholerae*, *Escherichia coli*, *Salmonella enterica* serovar Typhimurium, *Brucella abortus*, *B. suis*, *B. ovis*, *B. melitensis*, or *B. neotomae*. We tested the immunohistochemistry in cases of abortion caused by *Campylobacter jejuni* and *C. fetus* but observed no cross-reactivity. Although cross-reaction with other members of Francisellaceae cannot be completely ruled out, *F. tularensis* is currently the only species of this family recognized as an ovine abortifacient. Definite species and subspecies identification requires bacterial isolation and DNA analysis, which we were unable to perform because the available specimens were unsuitable.

Sheep with tularemia have been implicated in disease transmission to sheep industry workers (*10*). In the case described here, the owners of the sheep lived on the farm and were in contact with the affected flock regularly; however, we do not know whether they had clinical signs consistent with tularemia.

The source of infection in this sheep remained unknown. However, *F. tularensis* has a broad animal reservoir, including arthropods, rodents, lagomorphs, and marsupials (*6*,*14*). Brown hares (*Lepus europaeus*), a species that plays a primary role in the ecology of tularemia in Europe (*12*), have been introduced to Uruguay and are frequently seen around the affected farm. In addition, *F. tularensis* can be transmitted by ticks, several of which, including *Amblyomma* spp., *Haemophysalis* spp., and *Ixodes* spp. ticks, are endemic in Uruguay. Of note, a gamma-proteobacterium related to *Francisella*-like organisms, but different from *F. tularensis*, was identified in Uruguay in *Amblyomma triste* ticks (*15*), the most prevalent tick species reported in human tick bites in the country.

## Conclusions

We provide pathologic and immunohistochemical evidence of disease caused by a possible Francisellaceae member in sheep in Uruguay. Additional research is needed to isolate and speciate the pathogen and elucidate its regional epidemiology. Nonetheless, veterinarians, physicians, and public health officials should be aware of possible tularemia in South America. 

Appendix Additional information on the immunohistochemical procedure used to detect *Francisella* among aborted ovine fetuses, Uruguay.
