# Supplementary material for: Pathologic and Immunohistochemical Evidence of Possible Francisellaceae among Aborted Ovine Fetuses, Uruguay
Source: Emerg Infect Dis. 2023 Jan;29(1):141–4. doi: 10.3201/eid2901.220698 (PMC9796210; doi:10.3201/eid2901.220698)
Supplement: Appendix — Additional information on the immunohistochemical procedure used to detect Francisella among aborted ovine fetuses, Uruguay. [file 22-0698-Techapp-s1.pdf]

Article DOI: https://doi.org/10.3201/eid2901.220698

Pathologic and Immunohistochemical Evidence of Possible Francisellaceae among Aborted Ovine Fetuses, Uruguay

Appendix

Additional Methods

Immunohistochemical Procedure Used for Detection of *Francisella* Antigen in Formalin-Fixed Paraffin-Embedded Tissues

Heat induced antigen retrieval was performed in a decloaking chamber at 110°C for 10 min in Antigen Decloaker citrate buffer pH 6.0 (Biocare Medical, https://biocare.net). A blocking step for endogenous peroxidase was performed with 3% aqueous solution of hydrogen peroxide for 10 min. A specific mouse monoclonal IgG3 raised against *F. tularensis* lipopolysaccharide, *Francisella tularensis* LPS Monoclonal Antibody (T14) (Thermo Fisher Scientific, https://www.thermofisher.com), was applied as the primary antibody at a 1:1,000 dilution for 45 min at room temperature. Mouse-on-Farma horseradish peroxidase-labeled polymer (Biocare Medical) and 3-amino-9-ethylcarbazole (Thermo Fisher Scientific) was used as the detection method.
